# Low Serum Vitamin D Is Not Correlated With Myopia in Chinese Children and Adolescents

**DOI:** 10.3389/fmed.2022.809787

**Published:** 2022-02-04

**Authors:** Xiaoman Li, Haishuang Lin, Longfei Jiang, Xin Chen, Jie Chen, Fan Lu

**Affiliations:** ^1^The Eye Hospital, School of Ophthalmology and Optometry, Wenzhou Medical University, Wenzhou, China; ^2^Wenzhou Medical University, Wenzhou, China

**Keywords:** myopia, serum 25(OH)D, children, lowland, highland

## Abstract

**Purpose:**

This cross-sectional study investigated the association between serum 25-hydroxyvitamin D [25(OH)D] concentration and myopia in two groups of Chinese children aged 6–14 years from different geographic and economic locations.

**Methods:**

A total of 294 children from a lowland area and 89 from a highland area were enrolled as two groups of study subjects. The visual acuity, ocular biometry, and automated refraction were measured. The serum level of 25(OH)D was determined by chemiluminescence immunoassay. Near vision and outdoor exposure durations were assessed with a questionnaire interview. Data were analyzed for differences using Chi-square and Wilcoxon rank sum tests. The risk factors were evaluated using logistic regression analysis.

**Results:**

We found that the serum level of 25(OH)D of the subjects from lowland area was 20.9 ng/mL which was higher than that of subjects from highland area (16.9 ng/mL). The median spherical equivalent refraction (SER) was −0.25 diopters(D) in lowland subjects and −0.63D in highland subjects. The prevalence of myopia was 45.2% in lowland subjects and 55.1% in highland subjects. The average axial length was similar, 23.6 mm and 23.1 mm in lowland and highland subjects, respectively. We found no statistical difference between the average SER and serum 25(OH)D concentration in subjects of either lowland or highland area. The ratio of myopia to non-myopia was also similar in subjects with three levels (sufficient, deficient, and insufficient) of serum 25(OH)D in these two areas.

**Conclusions:**

There is no association between serum 25(OH)D concentration and myopia in the 6–14 years old Chinese children.

## Introduction

In the past 10 years, the prevalence of myopia has increased by 23% in East Asians (1). In Chinese children, the prevalence was 51.4% in Anyang (2), 34.9% in Guangzhou (3), 60.9% in Beijing (4) and these numbers are still rising (5). The prevalence of myopia in Haidian District, Beijing, increased from 55.9 to 65.5% from 2005 to 2015 (6). Myopia is now a worldwide public health problem affecting more and more young children (4, 7). However, the reasons for this multifactorial disease and its mechanism are still unclear (8, 9).

More outdoor exposure is known to slow the onset and progression of myopia (9–12). The seasonal effect on the progression of the axial length (AL) in children is confirmed. Increase in AL slows down with longer duration of sunlight exposure and high sunlight intensity (11, 13). Myopia progression is known to be slower in summer than in winter (14, 15). In Tibetan highlands, myopia prevalence is lower than that in lowland areas, presumably attributed to less education pressure, more sunlight exposure and higher sunlight intensity (16). The notion that sunlight exposure prevents myopia progression remains a controversy (17). Dopamine decrease AL growth is the most convincing hypothesis (18, 19), although there is not enough research in humans. A more uniform refraction pattern (20), longer depth of field and fewer high order aberrations (21) in an outdoor visual environment are factors that contribute to a sharper retina image, and these factors might contribute to slower AL growth.

The internal homeostasis of human body, especially serum vitamin D, is closely associated with outdoor activities. Serum vitamin D is composed of vitamin D_3_ and vitamin D_2_. Vitamin D_3_, the main source of vitamin D in the body, is formed in the skin though sunlight exposure. Vitamin D_2_ is absorbed through diary intake. Vitamin D_3_ and vitamin D_2_ are transformed into serum 25-hydroxyvitamin D [25(OH)D_3_] and 25(OH)D_2_, respectively, in the liver, and the serum level of 25(OH)D is the most reliable indicator of total vitamin D in the body (22).

Previous studies have investigated the relationship between the serum level of Vitamin D and myopia. Choi et al. investigated 2,038 adolescents aged 13 to 18 years old in Korea and found that serum 25(OH)D concentrations were 16.3 ± 0.3 ng/mL in the non-myopia group, 16.4 ± 0.3 ng/mL in the mild-myopia group, 16.0 ± 0.3 ng/mL in the moderate-myopia group, and 15.2 ± 0.4 ng/mL in the high-myopia group (*P* = 0.054) (23). A meta-analysis showed that the myopia group had lower 25(OH)D concentration than the non-myopia group (standard mean difference = −0.27 nmol/L, *P* = 0.001) (24). Researchers showed that lower Vitamin D is associated with increased risk of myopia (25–30). Besides refractive error, the change of AL was also reported in literature. A comprehensive cross-sectional study conducted by Tideman et al. in Netherlands showed that vitamin D was correlated with a longer AL in 6-year-old children (31). However, some of the other studies did not show any correlation between Vitamin D and refractive error (32–35). Hung-Da et al. found that the mean serum 25(OH)D concentrations were similar between the myopic and non-myopic groups (49.7 ± 13.6 and 48.8 ± 14.0 nmol/mL; *P* = 0.806) in 99 children with a mean age of 6.8 years (36). The prevalence of vitamin D deficiency and insufficiency is more than 80% in some age groups in China, a value much higher than those for other countries (37). Vitamin D deficiency in China is commonly seen even in developed regions like Guangzhou and Beijing (37–39). Whether vitamin D concentration, especially a relatively low concentration vitamin D, has an impact on the development of myopia is uncertain.

Vitamin D is generated in the body through light exposure and diary intake. In this study, we selected two groups of subjects from locations with substantially different diets and exposure to sunlight. Our intention was to test whether vitamin D is associated with myopia in these two groups of subjects with different levels of vitamin D concentration.

## Materials and Methods

### Study Population

The study subjects were children aged 6–14 years old in both lowland and highland areas. Lowland subjects were enrolled from the Health Examination Center of the Second Affiliated Hospital of Wenzhou Medical University (WMU) (Sept 2011–Mar 2016). Their counterparts were subjects from the Highland Eye Study conducted in Tibet by the Eye Hospital of WMU (July 2013 to Aug 2013). Their inclusion criteria were: (1) Aged 6–14 years. (2) No metabolic or congenital systemic diseases. (3) No eye diseases, such as cataract, glaucoma, amblyopia, and others. (4) No history of orthokeratology contact lens wear or any other myopia control treatments such as atropine within 3 months. (5) No mental illness. (6) Children and their guardians were able to understand the aim and procedures of the study and sign the informed consent forms. The study was approved by the Ethics Committee of the Eye Hospital of Wenzhou Medical University (KYK[2014]26). Written informed consent was obtained from all subjects prior to participating in the program. The study followed the tenets of the Declaration of Helsinki.

### Eye Examinations

Eye exams included slit lamp tests, refraction and biometry. Distance visual acuity was examined with an ETDRS visual chart at 4 m. Slit lamp was used to examine anterior eye structures. Auto-refraction (Topcon RT 8900) was tested several times until the readings of the refractive error became stable and the final reading was recorded. Biometry measurements were tested with Lenstar optical biometry (Lenstar 900). At least three stable tests were obtained and the readings of AL, anterior chamber depth, lens thickness, central corneal thickness, corneal diameter, corneal curvature, corneal astigmatism were recorded. If the subject and his/her guardian agreed to cycloplegia, 1% cyclopentolate hydrochloride would be used twice with a 5 min interval. Among 352 subjects in the lowland groups, 294 subjects (83.5%) who underwent cycloplegic refraction were included in the study. Subjects in the highland groups did not undergo cycloplegic refraction.

### Blood Sample Collection and Vitamin D Detection

Three milliliter- fast blood sample was drawn and kept away from light for 30 min, then centrifuged (3,000 r/min) for 10 min. The blood serum was separated and frozen at −80°C refrigerator for 25(OH) D detection. Total vitamin D assay kit (SIMENS,100T) was used to detect 25(OH)D through the automatic chemiluminescence immunoassay analyzer Chemiluminescence (SIMENS, ADVIA Centaur XP, IRL26391136).

### Questionnaire Interview

Demographic information, near vision duration, and outdoor duration were sought in the questionnaire. Near vision and outdoor duration were recorded for 3 conditions: on weekdays, during weekends and during summer/winter vacation.

### Definitions

Myopia was defined as non-cycloplegic spherical equivalent refraction (SER) ≤-0.50 diopters(D) (40). Serum 25(OH)D concentration higher or equal to 30 ng/mL was defined as a sufficient level. Concentrations less or equal to 20 ng/mL were defined as deficient serum 25(OH)D and the concentrations between these extremes were defined as insufficient serum 25(OH)D (41).

### Quality Control

Procedures for testing the subjects from both lowland and highland areas followed the same protocol. Questionnaire interviews were conducted by investigators who had good communication skills, knew local dialect and received training by the supervisors (CJ, LF) before the project began. A prior test was conducted to adjust improper procedures. The 25(OH)D detection was conducted by professional laboratory staff of the Eye Hospital of WMU.

### Statistical Analysis

SER, 25(OH)D, AL and age were described as median (interquartile range) and Wilcoxon *t*-test was used for comparisons. Chi-square test and Fisher exact test were used for classical variables. A multivariable logistic regression model was constructed to assess the association between myopia and 25(OH)D concentration and adjusting for age, gender, and other covariates identified as being significant in univariable analysis. *P* < 0.05 was considered as statistically significant. Because SER and AL of both eyes were highly correlated, only data of the right eye was used in the analysis. Epidata (version 3.1.2701.2008, Chinese) was used for double-blinded data entry. SPSS (version 22.0, Chinese) was used for analysis.

## Results

### Demographic Information and Myopia

A total of 383 subjects were enrolled in this analysis, including 294 lowland and 89 highland subjects. Compared with the lowland subjects, highland subjects were about 3 years older (12 vs. 9, *P* < 0.001), more girls (57.3 vs. 37.8%, *P* < 0.001), with lower BMI (16.7 vs. 17.4, *P* = 0.010), had longer duration for both near vision (28.0 vs. 16.6 h/w, *P* < 0.001) and outdoor activities (14.0 vs. 6.2 h/w, *P* < 0.001; Table 1). The median SER was −0.25 D in lowland subjects and −0.63 D in highland subjects (*P* = 0.163). Lowland myopia subjects were 2 years older than non-myopia subjects, with shorter outdoor duration (5.5 vs. 6.6 h/w, *P* = 0.009) and longer near vision duration (15.4 vs. 18.0 h/w, *P* = 0.010). Highland myopia subjects were 1 year older than non-myopia subjects, and their near vision and outdoor duration were similar. The AL of myopia subjects was 24.2 and 23.2 mm in non-myopia subjects in the lowland area (Table 1).

**Table 1:** Demographic information of subjects in lowland and highland area^*^.

	**Total**			**Lowland**			**Highland**		
	**Lowland**	**Highland**	***P***	**Non-myopia**	**Myopia**	***P***	**Non-myopia**	**Myopia**	***P***
	**(*n* = 294)**	**(*n* = 89)**		**(*n* = 161)**	**(*n* = 133)**		**(*n* = 40)**	**(*n* = 49)**	
Age (year)	9.4 (3.3)	12.0 (3.0)	<0.001	8.5 (2.7)	10.6 (2.7)	<0.001	12.0 (3.0)	13.0 (4.0)	0.456
**Gender**, ***n*** **(%)**									
Boys	183 (62.2)	38 (42.7)	<0.001	102 (63.4)	81 (60.9)	0.378	16 (40.0)	22 (44.9)	0.826
Girls	111 (37.8)	51 (57.3)		59 (36.6)	52 (39.1)		24 (60.0)	27 (55.1)	
BMI (kg/m^2^)	17.4 (6.1)	16.7 (3.6)	0.010	17.4 (5.9)	17.4 (7.4)	0.664	17.1 (3.1)	16.4 (4.9)	0.488
Outdoor time (h/w)	6.2 (5.9)	14.0 (7.0)	<0.001	6.6 (6.2)	5.5 (5.5)	0.009	–	–	
Near vision time (h/w)	16.6 (14.6)	28.0 (17.5)	<0.001	15.4 (13.8)	18.0 (15.3)	0.010	–	–	
25(OH)D (ng/mL)	20.9 (11.6)	16.9 (6.5)	<0.001	19.6 (12.2)	22.5 (11.2)	0.878	17.6 (8.8)	16.7(6.6)	0.216
Sufficient, *n* (%)	56 (19.0)	3 (3.4)	<0.001	33 (20.5)	23 (17.3)	0.158	1 (2.5)	2 (4.1)	0.295^†^
Insufficient, *n* (%)	100 (34.0)	22 (24.7)		47 (29.2)	53 (39.8)		13 (32.5)	9 (18.4)	
Deficient, *n* (%)	138 (46.9)	64 (71.9)		81 (50.3)	57 (42.9)		26 (65.0)	38 (77.6)	
SER(D)	−0.25 (1.7)	−0.63 (3.1)	0.163	0.25 (0.5)	−1.6 (1.9)	–	0.25 (1.3)	−2.5 (3.4)	–
Myopia, *n* (%)	133 (45.2)	49 (55.1)	0.066	–	–		–	–	
Non-myopia, *n* (%)	161 (54.8)	40 (44.9)		–	–		–	–	
AL (mm)	23.6 (1.3)			23.2 (1.0)	24.2 (1.1)	<0.001			

* *Median (interquartile range) was used to describe continuous variables. Chi-square test was used in classification variable comparisons. Wilcoxon rank sum test was used in continuous variable comparisons*.

† *Fisher's exact test*.

### Relationships Between Serum 25(OH)D and Myopia

**Figure 1:**
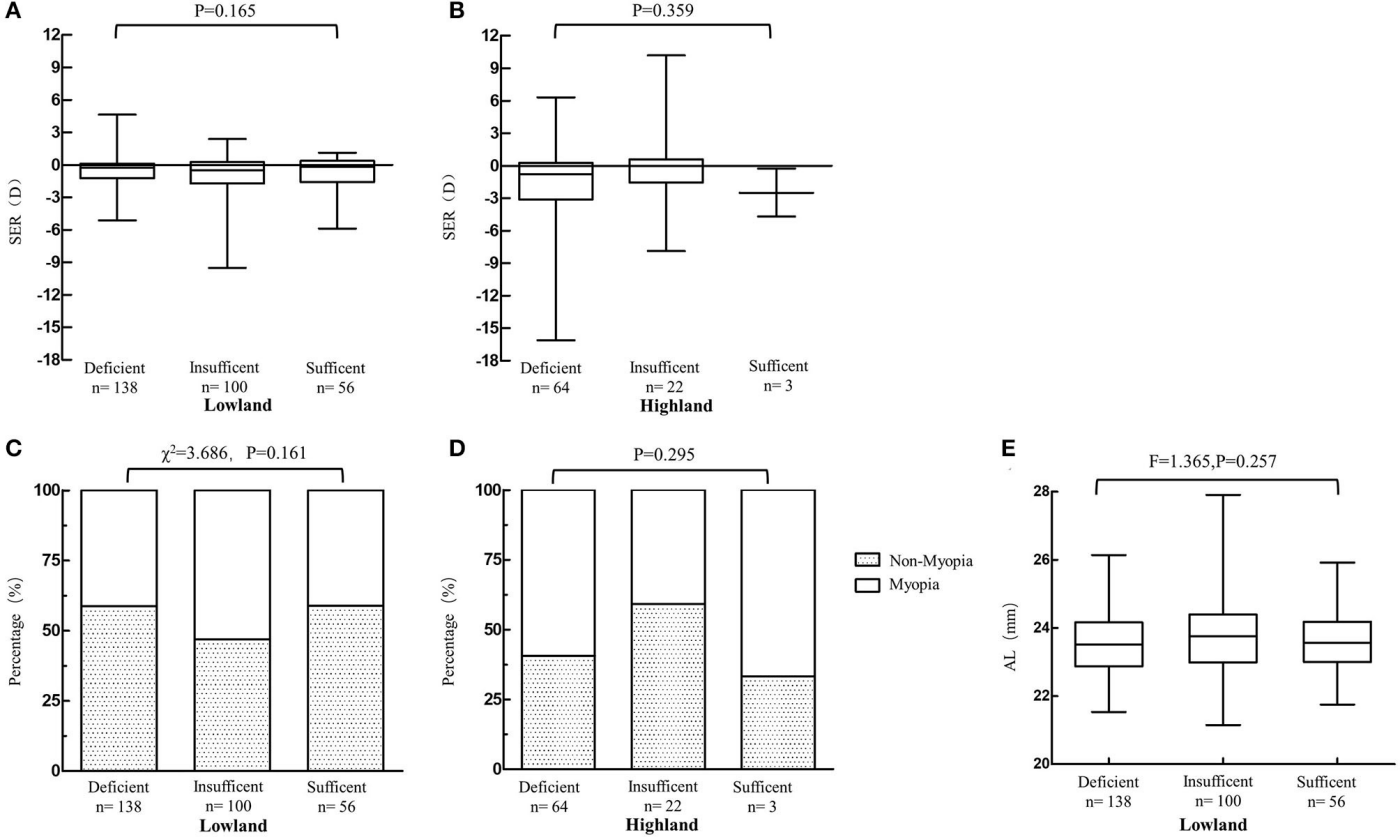
Myopia related parameters among groups with different serum vitamin D [25(OH)D] concentration in both areas. **(A,B)** Average SER among groups with different serum vitamin D concentration in both areas (Wilcoxon rank sum test). **(C,D)** Percentage of myopia and non-myopia among groups with different serum vitamin D concentration in both areas (Lowland: Chi-square test; Highland: Fisher exact test). **(E)** Axial length among groups with different serum vitamin D concentration in lowland subjects (Wilcoxon rank sum test).

The serum level of 25(OH)D in highland subjects was lower than that in lowland subjects (16.9 vs. 20.9 ng/mL, *P* < 0.001) and the rate of deficiency in highland group was higher than that in the lowland group (71.9 vs. 46.9%, Table 1). We found no difference in serum 25(OH)D concentration between myopia subjects and non-myopia subjects in either lowland or highland areas (lowland: 19.6 vs. 22.5, *P* = 0.878; highland:17.6 vs. 16.7, *P* = 0.216). There was no statistical difference in the percentage of subjects with sufficient, insufficient or deficient 25(OH)D in both myopia and non-myopia groups in both areas (lowland: *P* = 0.158, highland: *P* = 0.295) (Table 1). The average SER was similar among subjects with different levels of 25(OH)D concentrations in both areas (Figure 1A). The percentage of myopia and non-myopia also showed no difference among the three levels of 25(OH)D of subjects in either location (Figure 1B). When refractive error was considered in terms of AL, no significant difference was found. The results were the same in the lowland area (*F* = 1.365, *P* = 0.257) (Figure 1C). We obtained AL measurements for only 13 subjects in the highland group and only three subjects showed 25(OH)D higher than 30 ng/ml. The medians (interquartile) of AL in the deficient group (*n* = 8) and the insufficient group (*n* = 4) were 22.8(1.17) and 23.4(1.19) respectively (*P* = 0.214).

Multiple logistic regression analysis revealed that the serum level of 25(OH)D was not significantly associated with myopia in either lowland area (*P* = 0.706) or highland area (*P* = 0.141) after adjusting age, body mass index (BMI) and gender (Table 2). Older age (odds ratio [OR]: 1.72; 95% confident interval [CI]: 1.46, 2.03) was an independent risk factor for myopia in lowland subjects. A higher BMI [OR(95%CI = 0.94(0.88, 1.00)] seems to be a protective factor for myopia. For the highland subjects, none of the factors mentioned above was statistically significant in multiple logistic regression analysis. The results were similar to the lowland group when all subjects were included. Older age [OR(95%CI) = 1.55(1.36–1.76)] and living in lowland area [OR(95%CI) = 2.13(1.10, 4.15)] were independent risk factors of myopia after adjustment, whereas higher BMI [OR(95%CI) = 0.94(0.88, 0.99)] was independent protective factor (Table 2).

**Table 2:** Multiple logistic regression of the myopia risk factors in lowland and highland area.

**Factors**	**Lowland^†^**		**Highland^‡^**		**Total^§^**	
	**OR (95%CI)**	***P***	**OR (95%CI)**	***P***	**OR (95%CI)**	***P***
Age (year)	**1.72 (1.46–2.03)**	**<0.001**	1.13 (0.90–1.41)	0.306	**1.55 (1.36–1.76)**	**<0.001**
**Gender**						
Boys	Reference		Reference		Reference	
Girls	1.30 (0.73–2.32)	0.373	1.02 (0.39–2.65)	0.969	1.20 (0.75–1.91)	0.456
BMI (kg/m^2^)	0.94 (0.88–1.00)	0.066	0.90 (0.76–1.06)	0.208	**0.94 (0.88–0.99)**	**0.029**
25(OH)D (ng/mL)	1.01 (0.98–1.04)	0.706	0.94 (0.87–1.02)	0.141	0.99 (0.97–1.02)	0.676
Outdoor time (h/w)	0.95 (0.90–1.01)	0.120	–	–	–	–
Near vision time (h/w)	1.02 (0.99–1.04)	0.273	–	–	–	–
**Region**						
Highland	–	–	–	–	Reference	
Lowland	–	–	–	–	**2.13 (1.10–4.15)**	**0.026**

*OR with statistically significance were listed in bold*.

† *Adjustment for age, gender, BMI, outdoor time and near vision time*.

‡ *Adjustment for age, gender, and BMI*.

§ *Adjustment for age, gender, BMI and region*.

The SER of lowland subjects before and after cycloplegia was −0.78 ± 1.68D and −0.26 ± 1.85D, respectively, a difference of about a 0.5D. In order to know whether cycloplegia could affect the accuracy of these results, we conducted the same analysis using cycloplegic refraction data for our lowland group. Results were similar as using non-cycloplegic SER. These results demonstrated that non-cycloplegia had no confounding effect on the regression models.

## Discussion

The results in our study revealed that the serum vitamin D concentration does not show any protective effect toward myopia. Outdoor exposure is one of the protective factors of myopia, but its mechanism is controversial (17). One hypothesis is that vitamin D prevents myopia progression because the body synthesizes more vitamin D when outdoor exposure to sunlight increases (26). Our study involved subjects from a relatively high vitamin D concentration area and a relatively low vitamin D concentration area, yet results showed that serum vitamin D was not associated with either myopia or AL. The results of our study was similar to previous studies such as meta-analyses and prospective cohort studies (33–35). Neither vitamin D_3_ which formed from sunlight in the skin nor vitamin D_2_ from diary intake correlated with myopia, however, serum level of vitamin D was positively correlated with outdoor duration. There were also opposite results showing that subjects with higher vitamin D had shorter AL or positive SER (24, 31, 42).

In this study, the incidence of insufficient and deficient serum vitamin D of children was similar to or higher than that found in other studies (Table 3) (38, 39, 43–48). Serum vitamin D was 20.9 ng/mL in lowland subjects and 16.9 ng/mL in highland subjects. Only 19.0 and 3.4% of these subjects had optimal vitamin D level in lowland and highland areas, respectively. Thus, whether serum vitamin D didn't show its protective effect on myopia because of a relatively low concentration is worth to thinking about. To fully consider this possibility, two locations were chosen to understand the relationship between myopia and vitamin D. Subjects in highland areas came from Seda, Litang and Rangtang, small towns in the Tibetan highland, Sichuan, where both daily sunlight duration and sunlight intensity are higher than that of lowland area (Table 4). And serum vitamin D was not related to SER nor AL in either area. This result indicated that maybe for Chinese children, serum vitamin D concentration does not show any association with myopia.

**Table 3:** Children's vitamin D level in different regions of China.

**Year**	**Region**	**Age**	**Detection method**	**Detection rate of Sufficiency %**	**Mean ± SD (ng/mL)**
2011–2013	Guangzhou (37)	0–14	ELISA^†^	36.6	28.3 ± 8.4
		7–14		11.5	23.0 ± 5.9
2012–2013	Beijing (39)	0–14	MS-MS^‡^	12.9	22.1 ± 8.5
2017	Jiaxing of Zhejiang (43)	3–6	MS-MS	–	23.0 ± 7.7
2013	Shaoxing of Zhejing (44)	0–9	CLIA^§^	45.8	29.8 ± 12.8
2017	Shanxi (45)	9–16	CLIA	34.6	18.0 ± 6.4
2012	Guangxi (46)	6–13	ELISA	22.5	22.9 ± 0.4
2017–2018	Mianyang of Sichuan (47)	0–8	MS-MS	17.1	18.6
2016	Shenmu of Shanxi (48)	0–14	ELISA	14.8	13.3 ± 6.0
2015–2016	Heilongjiang (49)	0–6	MS-MS	–	24.2 ± 10.0
2011–2016	Wenzhou of Zhejiang^*^	6–14	CLIA	19.0	22.3 ± 8.9
2013	Tibetan Highland^*^	6–14	CLIA	3.37	18.2 ± 6.0

† *ELISA stands for enzyme-linked immunosorbent assay*.

‡ *MS-MS stands for tandem mass spectrometry*.

§ *CLIA stands for chemiluminescence immunoassay*.

* *Children in our study*.

**Table 4:** Comparisons of regional characteristics of lowland and highland area.

**Characteristics**	**Lowland**	**Highland**
Location	Southeast of Zhejiang Province, China. Lowland area.	Northwestern of Sichuan Province, Tibetan Plateau in Southwest China.
Altitude	10 meters.	More than 3,300 meters.
Climate	Central Asian tropical monsoon climate zone, with four distinct seasons and abundant rainfall.	Continental plateau monsoon type. No summer. Frost and snow occur in fourth seasons, and the atmospheric oxygen content is <60% of the standard.
Sunshine	Annual sunshine between 1,442 and 2,264 h.	Average annual sunshine about 2,451.1 h.
**Time of sunrise and sunset**		
Summer solstice	Sunrise at 5:01 sunset at 18:56.	Sunrise at 6:12 sunset at 20:28.
Winter solstice	Sunrise at 6:44 sunset at 17:06.	Sunrise at 8:16 sunset at 18:18.
Temperature	Annual average temperature is 17.3–19.4°C. Average temperature in January is 4.9–9.9°C. Average temperature in July is 26.7–29.6°C.	Annual average temperature is −0.16°C. Average temperature in January is −11.1°C. Average temperature in July is 9.9°C.
Rainfall	Annual precipitation between 1,113 and 2,494 mm. Raining season comes at the end of spring and early summer. Tropical cyclone happens between July and September. Frost-free period is 241–232 days.	Average precipitation 65.4 mm, and mostly happens during June to September. Average frost-free period is 21 days. Areas with higher altitude has no absolute frost-free period.

Although average daily sunshine duration is longer, the light intensity is higher in highland area, and highland subjects spent more time outdoors, their average total vitamin D level was lower than that in lowland subjects which was quite surprising. This phenomenon might be due to an increase in the amount of skin pigment and Tibetan robes covering most of the skin of the body, that may result in an increase in the sun-protection permitting less of the UVB radiation to reach the epidermal cells, and reduce cutaneous production of vitamin D3 (50). In addition, it is speculated that rare vitamin D_2_ from daily intake may also contribute to the lower total vitamin D level of highland subjects. The three highland area are all National-level Poor Counties in China. Residents living here mainly live on Zanba (made of a cereal power specially seen in Tibetan highland areas), milk tea, handmade yogurt and yak meat. These foods comprise 90% of their daily intake, and deep sea fish and animal viscera are very uncommon in their diet. Tibetan Buddhism is a prevailing religion in Tibetan highland areas and many of the residents are vegetarians who do not eat yak meat. Our lowland area was Wenzhou city, a coastal city with abundant rainfall in eastern China (Table 4). Subjects of Wenzhou came from a health examination center and patients with any health problems were excluded from participation. The average family income of these Wenzhou children was higher than the per capita income of urban residents in Wenzhou in 2016 (51). The mean BMI was higher in these lowland subjects than highland counterparts (17.4 vs. 16.7, *P* = 0.010, Table 1). It could be speculated that the nutritional status of the subjects in the lowland area were better than those of their highland peers. These two reasons may explain why serum vitamin D was lower in highland subjects.

The prevalence of myopia in China is reported to be lower in Tibetan highland areas than lowland areas (52). The reason why myopia rate in the highland area was higher than lowland area may be because of age. The subjects in our highland areas are on average 3 years older than lowland subject. Age as a contributing factor of myopia must be considered in the group comparisons. The lowland subjects with an average age of 9 years old probably were in the third grade. The highland subjects with an average age of 12 years old were probably in the sixth grade. The myopia of grade 6 students was about 65% in lowland areas (53), which is much higher than the highland results in this study.

Duration of near vision and outdoor activity did not show a relationship with myopia compared with results from previous studies. Perhaps this is because of our highly selected samples. Lowland subjects were recruited from a health examination center where 74.2% of them were studying in downtown schools or key elementary schools, schools that demanded more homework and extracurricular tutoring. Most highland subjects were Buddhists in a Buddhist college with daily intensive reading courses. From Supplementary Figure, the distributions of both near vision and outdoor duration show high kurtosis and skewness. A significant correlation with myopia is difficult to obtain given the small sample size. Literatures reported that outdoor time is not significant risk to myopia. And the explanation is that the outdoor time per day was far <2 h/day and could not show its protecting effect (54, 55). This was exactly the fact in this study.

The merits of our study were the selection of subjects from two regions with significant contrasts, taking sunlight exposure and diary intake into account, and both refraction and AL were measured. The limitations were that the sample in our highland group was not large enough. A validated sun exposure questionnaire was not used to account for factors such as sun cream used and clothing worn. There are many other environmental and economical difference between lowland and highland areas that may impact the prevalence of myopia and should be considered in future studies. Compared to highland area, lowland area was risk toward myopia in this study. This maybe because subjects on the highland areas were from rural areas, the multi-effect of education pressure and modern urbanization affected the progression of myopia (52, 56, 57).

In summary, the total serum vitamin D concentration has no significant effect on myopia in Chinese children and adolescents. The mechanism of outdoor exposure affecting the progression of myopia should be further explored.

## Data Availability Statement

The original contributions presented in the study are included in the article/supplementary material, further inquiries can be directed to the corresponding authors.

## Ethics Statement

The studies involving human participants were reviewed and approved by Ethics Committee of the Eye Hospital of Wenzhou Medical University. Written informed consent to participate in this study was provided by the participants' legal guardian/next of kin.

## Author Contributions

XL contributed to the formal analysis and original draft writing. HL contributed to the investigation, formal analysis, validation, and data curation. LJ contributed to the data collection. XC contributed to the investigation. JC contributed to the investigation, resources, and draft revision. FL contributed to supervision, methodology, funding acquisition, and draft revision. All authors contributed to the article and approved the submitted version.

## Funding

This study was funded by the National Key Research and Development Program of China (2020YFC2008200), the Natural Science Foundation of China (Grant No. 81570880), and the Wenzhou Science and Technology Bureau Basic Research Funding (Grant No. Y20190166).

## Conflict of Interest

The authors declare that the research was conducted in the absence of any commercial or financial relationships that could be construed as a potential conflict of interest.

## Publisher's Note

All claims expressed in this article are solely those of the authors and do not necessarily represent those of their affiliated organizations, or those of the publisher, the editors and the reviewers. Any product that may be evaluated in this article, or claim that may be made by its manufacturer, is not guaranteed or endorsed by the publisher.

## Supplementary Material

Supplementary Figure The distribution of near vision time and outdoor time. **(A)** The distribution of near vision time in lowland area. **(B)** The distribution of near vision time in highland area. **(C)** The distribution of outdoor time in lowland area. **(D)** The distribution of outdoor time in highland area.
