# Supplementary figures and images for: Low Serum Vitamin D Is Not Correlated With Myopia in Chinese Children and Adolescents
Source: Front Med (Lausanne). 2022 Feb 4;9:809787. doi: 10.3389/fmed.2022.809787 (PMC8856508; doi:10.3389/fmed.2022.809787)

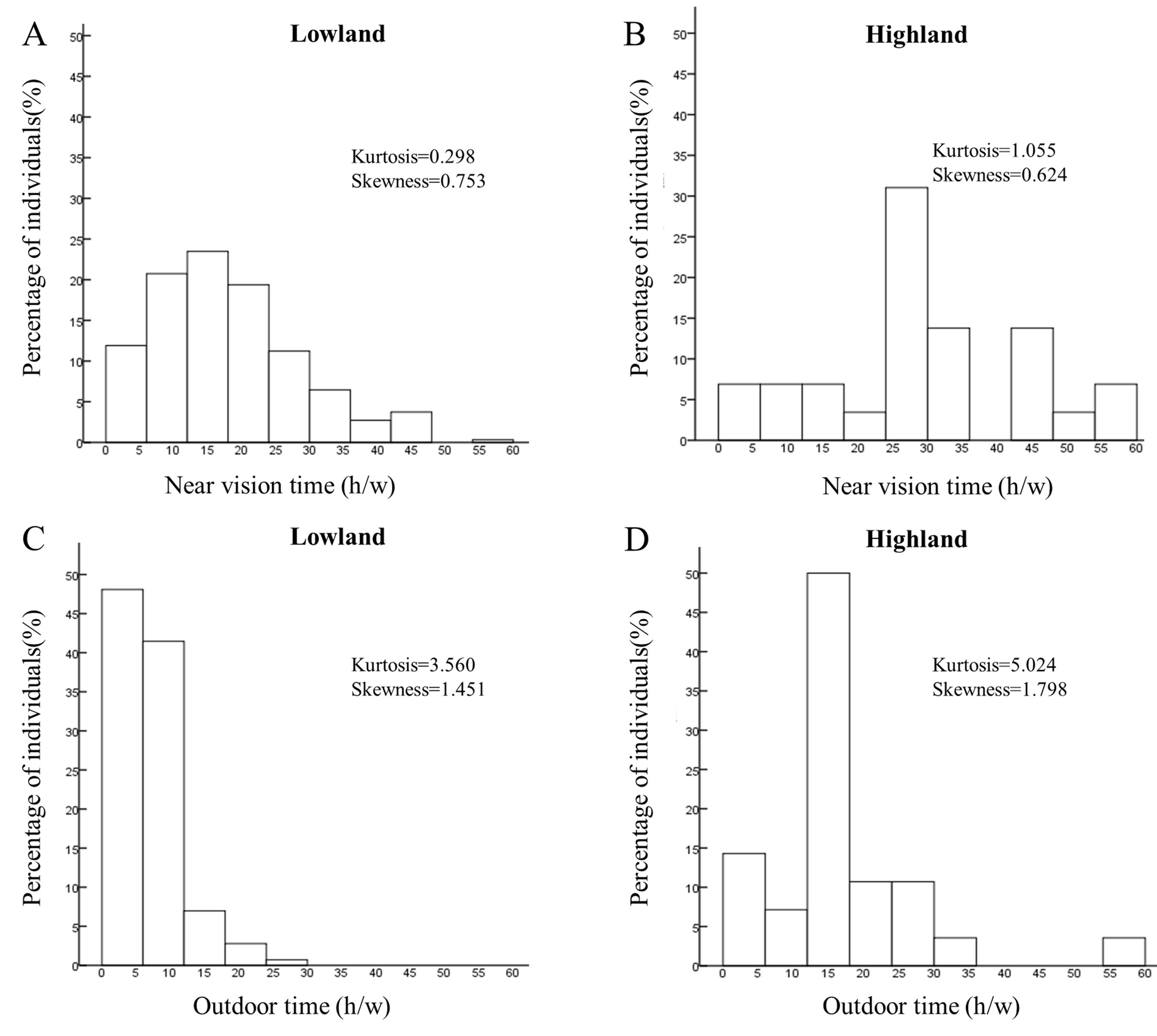

Supplement: Supplementary Figure — The distribution of near vision time and outdoor time. (A) The distribution of near vision time in lowland area. (B) The distribution of near vision time in highland area. (C) The distribution of outdoor time in lowland area. (D) The distribution of outdoor time in highland area. [file Image_1.jpeg]
